# Peculiarities in the Material Design of Buckling Resistance for Tensioned Laminated Composite Panels with Elliptical Cut-Outs

**DOI:** 10.3390/ma11061019

**Published:** 2018-06-15

**Authors:** Aleksander Muc

**Affiliations:** Institute of Machine Design, Cracow University of Technology, ul. Warszawska 24, 31-155 Kraków, Poland; olekmuc@mech.pk.edu.pl; Tel.: +48-12-6283353; Fax: +48-12-6283360

**Keywords:** composite laminated plates, buckling, optimal design, finite element analysis, elliptical cut-outs, continuous optimization, discrete optimization

## Abstract

The results of analytical and numerical studies of the buckling behavior of laminated multilayered tensioned sheets with circular and elliptical openings are presented. The analysis shows the significant influence of stress concentration effects on buckling modes and loads, particularly taking into consideration variations in the *E*_1_/*E*_2_ and *E*_1_/*G*_12_ ratios. The results of finite element (FE) computations prove that the buckling mode cannot be described by a single buckle localized at the apex of the hole. The optimal design of such structures seems to be much more complicated than classical buckling problems of compressed laminated panels without holes. However, the obtained results indicate that the optimal laminate configurations occur at the boundaries of the feasible regions of the introduced design space. Both continuous and discrete fibre orientations are considered. For continuous fibre orientations, the optimal stacking sequence corresponds to angle-ply symmetric laminates.

## 1. Introduction

The strength and buckling behaviors of laminated composite plates subjected to in-plane loads are important aspects in the preliminary design of aircraft and launch vehicle components. Holes are provided either in the center or elsewhere in the laminar plates for pipes for electric cables or other purposes. Due to the presence of holes in the plates, stress is concentrated near to the holes, and the stiffness of the plates is significantly reduced. In addition, a variety of modes of static and dynamic behavior are possible, and failures often result from the development of fatigue cracks which propagate from a stress concentration at the cut-out. The early solutions for the stresses in tensile sheets with circular holes obtained by Kirsch [1] in1898 or for elliptical cut-outs clearly [2] showed the regions of compressive stresses and stress concentrations. Then, Lechnitski [3] and Savin [4] demonstrated the existence of the same problems for anisotropic plates with holes ofdifferent shapes.

Various attempts have been made to reduce or eliminate stress concentrations. In general, they can be classified in three groups [5]. In general, the design of composite structures is directly connected to searching for *the material distributions* including (1) the *sizing* (addressed commonly to the choice of a thickness distribution around holes) [6,7], (2) the *shape optimization of cut-out* (the design of the domain of the design model) for plates [8,9] and cylindrical panels [10] and (3) the *topology* (understood in the sense of fibre orientations and/or fibre distributions; see, e.g., Gurdal et al. [11,12,13], Hyer et al. [14,15] and Parnas et al. [16]). Of course, each of the above problems can be formulated and solved separately. However, the nature of composite materials (two-phase materials with arbitrarily chosen fibre orientations—woven rovings and/or varying fibre volume fractions) and their layered structure allows us to join sizing, topology, and shape design in order to produce a new composite material that satisfies our specific mechanical and technological requirements (objective functions) of anisotropic plates with holes. For instance, each of the elements in the plated structure may have different thicknesses, volume fractions, shapes of reinforcement, mechanical properties and various fibre orientations, whereas shape variation is equivalent to the choice of elements having, e.g., thickness or mechanical properties equal to zero. The latter problem involves the determination of features, such as the number and location of holes and the connectivity of the domain.

**Figure 1:**
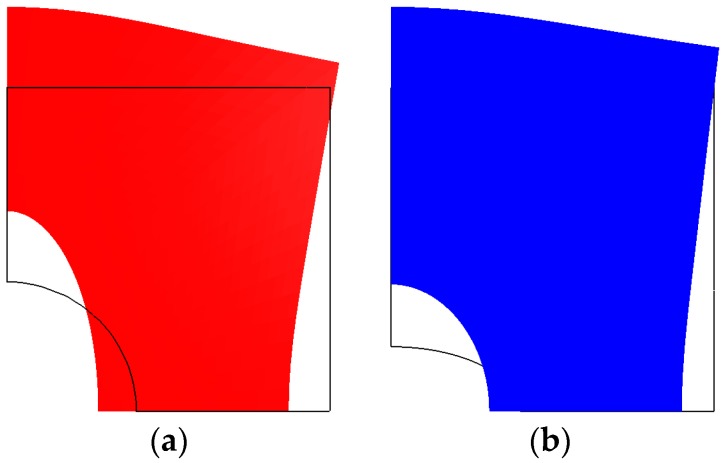
Distributions of total displacements around a hole for isotropic plates determined by finite element analysis: (**a**) circular; (**b**) horizontal ellipse (the scaling factor is 5, i.e., the computed values are multiplied by the factor 5).

It is well-known that for compressed structures, buckling is one of the possible failure modes. For the first time, in 1963, Cherepanov [17] described and analysed the buckling problem for stretched plates with a hole. If a plate contains a cut-out, regions of compressive stresses arise under a uniaxial tensile load. Figure 1 shows the compression effects due to tension for various shapes of holes.

In the past, the main focus has been on isotropic materials. The results of semi-empirical analyses of the crack/cut-out buckling problem have been presented in a number of works [18,19,20,21,22]. The empirical formula for the buckling stress of isotropic plates is expressed as

(1) $$\frac{N_{x}}{t}=3.55E{\left(\frac{t}{r}\right)}^{2},$$

where *E* is the Young’s modulus, *t* is the sheet thickness, and *r* is the hole radius. Shimizu [23] proposed the application of the Euler’s beam formula with a correction coefficient, *K_S_*:

(2) $$\frac{N_{x}}{t}=K_{S}\frac{\pi^{2}E}{12(1-\nu^{2})}{\left(\frac{t}{L_{x}}\right)}^{2}.$$

The buckling coefficient is derived numerically, and it is a function of the aspect ratio, *α* = *L_x_*/*L_y_*, and of the type of the hole (i.e., circular, square, etc.).

Only a few papers have taken the orthotropic behavior of composite plates into account [24,25,26]. Kremer and Schurmann [26] indicated that the initiation of buckling could precede fracture due stress concentrations and result in the final failure (modes and loads) under both static and fatigue loading conditions.

A broad review of the works that have dealt with the buckling behavior of compressed laminated plates and cylindrical shells with holes was done by Muc et al. [27] (two sections of the work) and therefore, this is not repeated and discussed herein.

This study is important in order as it aims to determine the buckling behavior of multilayered laminated plates with holes. With the aid of the finite element method (FEM) NISA II (v.19, EMRC, Troy, MI, USA), one is able to estimate buckling loads with respect to the orthotropic properties of the plate, but no analytical solution has been found so far, even for plates with circular holes.

One objective of the present study is to present an overview of past research, focusing on the identification of the analytical approaches used and then, describing the particular aspects of the behavior investigated. Then, attention is focused on the possible maximization of buckling loads with respect to fibre orientations (so-called topology optimization). The optimization results are demonstrated and verified with the aid of finite element computations in conjunction with the definition of new, specific design variables. Another objective is to determine key behavioral characteristics and trends arising in the buckling analysis of a rectangular laminated plate with an elliptical cut-out that address specific issues, such as the effects of plate anisotropy (laminate material properties), stacking sequences, and cut-out shape. Last, some closing comments about the obtained results and future works are given.

## 2. Formulation of the Buckling Problem and Derivation of Buckling Loads—The Rayleigh–Ritz Method

**Figure 2:**
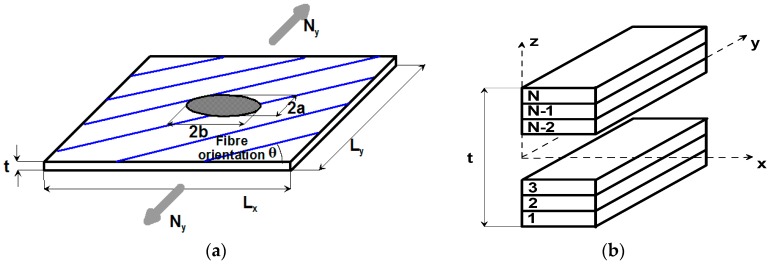
(**a**) The panel and the elliptical hole geometry; (**b**) stacking sequence, symmetric case.

To structurally model a plate subjected to bi-axial compression/tension, it is assumed that the coordinate system origin is located at the plate geometrical center on the mid-plane (Figure 2). It is also assumed that the plate is made of N layers where each of the plies has an identical thickness *t*/*N* (*t* is the total thickness of the panel and *t*/min(*L_x_*, *L_y_*) << 1). Each layer is made ofan identical, unidirectional, composite material. The plate is enforced to be symmetric about its mid-plane, requiring only half of the layers (i.e., *N*/2) to be designed. In addition, the plate is also required to have a balanced stacking sequence.

When a flat plate is subjected to an in-plane load, it initially remains flat and stays in equilibrium condition. However, as the in-plane force increases to a certain amount, the plate becomes unstable, and its configuration changes from flat to non-flat. The load at which the plate leaves its equilibrium condition and becomes unstable is known as the “*buckling load*”.

The linear buckling analysis of multilayered composite plates makes it possible to accurately determine the critical loads which are of practical importance in the stability analysis of thin plates. It shows the effects of different cut-out shapes, material properties, orientations of layers and the length/thickness ratio on the critical load.

The critical load multiplier, *λ*, can be obtained by imposing the stationariness (which corresponds to a minimum condition) of the total potential energy change, Π, at the critical buckling state:

(3) $$\Pi =\int_{\Omega }dxdy\left(D_{11}\varepsilon_{xx}^{2}+2D_{12}\varepsilon_{xx}^{}\varepsilon_{yy}^{}+D_{22}\varepsilon_{yy}^{2}+4D_{66}\varepsilon_{xy}^{2}\right)-\lambda \int_{\Omega }dxdy\left(N_{x}w_{,x}^{2}+N_{y}w_{,y}^{2}\right)$$

where Ω denotes the 2D space occupied by the mid-plane of the laminate; *D*_11_, *D*_12_, *D*_22_, *D*_66_ are the bending and in-plane shear stiffnesses (their explicit form is presented in the Appendix A); and *w* is the normal deflection of the plate. Note that Relationship (3) is written in the local Cartesian system of coordinates, where 1 corresponds to the fibre direction, and 2 corresponds to the perpendicular one.

In open literature and standard texts, buckling loads are often expressed using approximate simple formulae and design charts to aid designers in estimating the buckling strength of structural members. It is still necessary, however, for designers to perform the buckling analysis if more accurate results are required or if there is no standard solution available. There are a large number of techniques available that are used to evaluate buckling loads. Among them, we would like to point out three commonly used approaches.

*The finite element method.* The buckling nodal displacements are approximated by the shape functions at each of the finite elements describing the structure. The stationarity requirement (i.e., *Π* = 0) leads to a homogeneous system of equations for the load factor *λ*.

*The Bubnov–Galerkin method.* The buckling displacements are approximated by a series of functions with unknown coefficients, *c_ij_*, satisfying the boundary conditions. The homogeneous system of equations, obtained by the differentiation, $\partial \Pi /\partial c_{ij}=0$, allows buckling loads to be found.

*The Rayleigh–Ritz method.* The Rayleigh quotient is used in the min-max theorem to get exact values of all eigenvalues. It can be also used in eigenvalue algorithms to obtain an eigenvalue approximation from an eigenvector approximation. Specifically, this is the basis for the Rayleigh quotient iteration. A judicious choice for the trial function that satisfies kinematic boundary conditions and depends on the set of variational parameters must be given in advance. Therefore, the Rayleigh–Ritz variational principle is a powerful technique for the approximate solution of eigenvalue problems where a trial function (or functions) is introduced. The solution obtained from Equation (3) is an upper bound one when compared to exact solutions.

**Figure 3:**
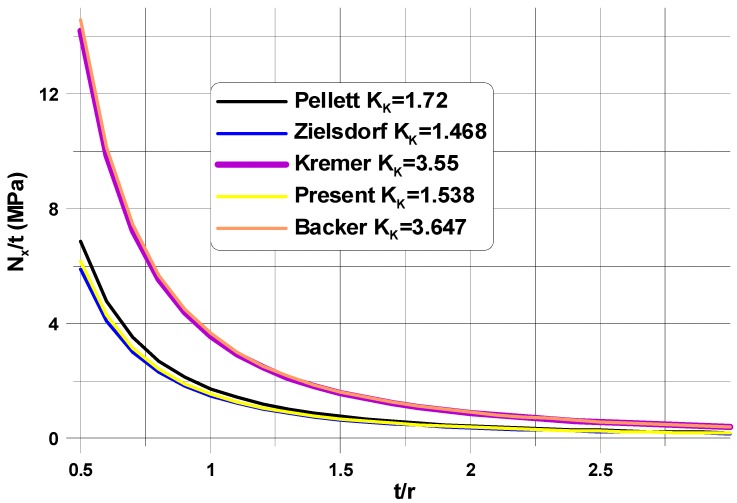
Comparison of the analytical buckling load predictions.

In the literature [28,29,30], analytical (mathematical) investigations of the local static stability of infinite isotropic plates with circular/elliptical openings subjected to a uni-axial tension loading have been conducted using the Bubnov–Galerkin or the Rayleigh–Ritz method. The buckling modes have been described with the help of expansion into series in the polar or elliptical systems of coordinates. The results of these studies were strongly affected by the number of terms in the expansions (see the comparison shown in Figure 3). The results obtained by the author (“present”) were derived with the use of one term of expansion. The correctness of the estimations is verified in Section 4.

## 3. Optimal Design

**Figure 4:**
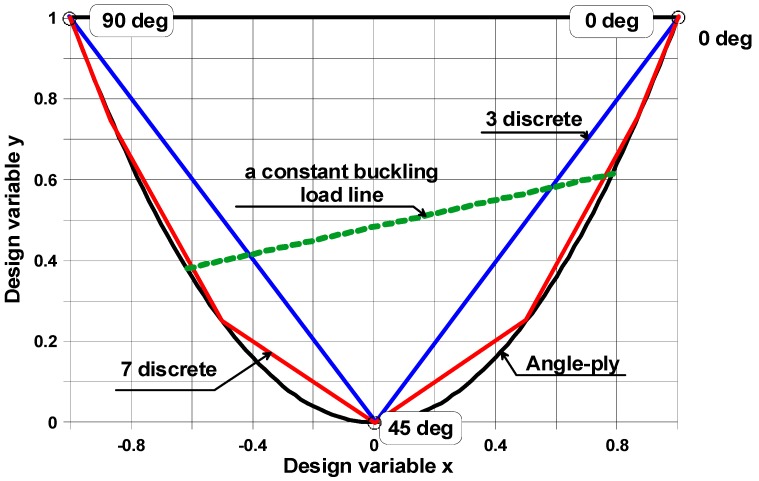
The design space for continuous and discrete design variables.

In order to find the laminate stacking sequence that is best suited to the load and geometrical boundary conditions under consideration, the ply orientations of the laminate as well as the ply thicknesses need to be used as design variables. For the laminate with N plies, the total number of design variables is equal to 2*N. It is impossible to solve such a problem analytically, and the numerical solution is troublesome since a lot of local minima and maxima exist. Therefore, we propose the use of the graphical optimization method to solve the optimization problem, which can be formulated in the following way to maximize the buckling load, expressed by Relationship (3) with respect to the 2*N design variables mentioned above. It is well-known that the design space is represented by the interior and the boundary of the parabola (Figure 4).

The axes of the coordinate system (*x*, *y*) are defined in Appendix A. Each point of the interior of the parabola is characterized by two real numbers that represent the laminate stacking sequence. The boundary of the parabola described by the function *y* = *x*^2^ corresponds to angle ply symmetric fibre orientations *±θ*, and in this way, for a laminate made of plies with identical thicknesses, the total number of variables is reduced to one variable, i.e., fibre orientation (*θ*). The diagram in Figure 4 can be used to design laminates with predetermined ply orientation angles (i.e., with discrete ply orientations). The feasible region for laminates with fixed ply angles is a polygon with vertices located on the envelope (the parabola). The possible forms of the polygons are drawn in Figure 4. For laminates with 0°, ±45°, and 90° plies, the design space is a triangle. For laminates with 0°, ±15°, ±30°, ±45°, ±60°, ±75°, and 90° plies, the space forms a polygon.

For uni-axially or bi-axially simply-supported, compressed plates, the trajectories characterizing the constant buckling loads constitute straight lines (Figure 4). In such a situation, the optimal stacking sequences cannot be determined uniquely since they correspond to the set of points (*x*, *y*) belonging to the portion of the straight line cutting the feasible region (see Muc [31,32]).

**Figure 5:**
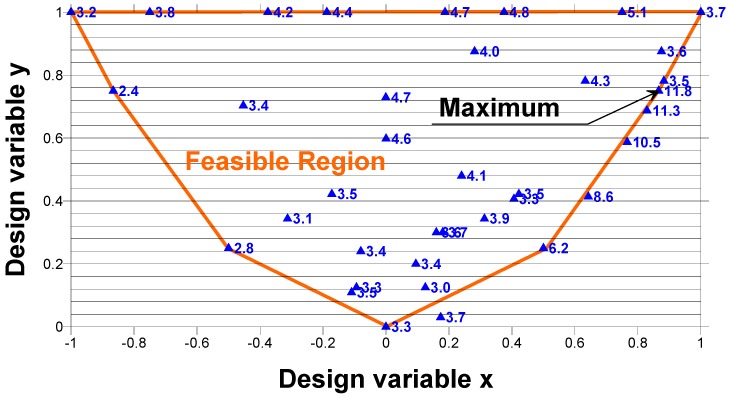
Post map of buckling loads in kPa—the square plate with the circular hole (2*b*/*L_x_* = 0.4); the orange lines denote the polygon for seven discrete fibre orientations.

The buckling analysis of laminated tensioned plates with cut-outs shows the opposite effect to that observed previously, i.e., the position of the maximal buckling load on the design space (*x*, *y*) is strictly localized and reduced to a point (Figure 5). The maximum occurs at the edge of the feasible region—the parabola for continuous fibre orientations or the polygon for a discrete set of allowable fibre orientations. Buckling of a structure is dominated by a change in the membrane stress state to a bending dominated stress state. The buckling load of composite plates is dominated by the local bending (*D*_11_, *D*_22_) and in-plane shear (*D*_66_) stiffnesses (Equation (3)) which depends on the spatial direction, the stacking sequence, and the fibre orientation.

## 4. Parametric Investigations

In regard to laminated plates weakened by holes, the problems associated with the finite element (FE) description and the analysis of the accuracy have been discussed in detail by Muc [8]. In general, the mesh division is controlled by two variables in the polar coordinate system: the radial distance from the hole and an angle measured from the x reference axis. The direction defined by the angle is controlled by a few parameters (exponential splines) in order to obtain predominantly equal curvature along the curve defining the opening. However, in the case of an increasing stress concentration, the amount of FE grows. Convergence tests are carried out with regard to the plane strain energy density (*U*) variations along the curve of the hole. The energy density (*U*) takes a form that is analogous to the first part of Equation (3) after the replacement of the bending stiffness (*D_ij_*) by the plane stiffnesses (*A_ij_*) and the parameters of curvatures by the in-plane strains. In the radial direction, the node concentration is controlled by two parameters enabling the geometric progression to be obtained.

The buckling loads are estimated with the use of the classical linear stability analysis. Since the FE package, NISA II, currently has four methods for eigenvalue extraction, the correctness of the evaluation of buckling loads can be determined by the comparison of the computed values for different methods. The FE analysis is based on a rectangular 2D mesh generated automatically with the aid of the rules described above. In the analysis, the FE are shaped as a four-noded quadrilateral. Each of the elements consists of a number of layers of perfectly bonded orthotropic materials. The nodes have six degrees of freedom (NKTP 32—the name of FE in the NISA II program), i.e., the transverse shear effects are included in the analysis.

Tan [33] derived several formulas to determine the stress concentration factor for an orthotropic panel subject to uni-axial tension with an elliptical hole (Figure 2). For infinite panels, with respect to the material constants, the stress concentration factor can be expressed in the following way:

(4) $$K_{t\infty }=\frac{\sigma_{y}\left(x=b,y=0\right)}{N_{y}/t}=1+\frac{b}{a}\sqrt{2\left(\sqrt{\frac{E_{x}}{E_{y}}}-\nu_{xy}+\frac{E_{x}}{2G_{xy}}\right)}.$$

To determine the finite dimensions of the plate, Tan [33] also proposed various correction factors. However, the above formulae cannot be applied directly to the buckling analysis because it does not take the bending effects into account (Equation (3)). It demonstrates only that the stress concentration effects are not only the function of Young’s moduli but also, of the in-plane Kirchhoff’s modulus (*G_xy_*) and Poisson’s ratio (*ν_xy_*). The effects of the Poisson’s ratio on the buckling loads were investigated by Seif and Kabir [34].

### 4.1. Influence of Mechanical Properties

**Figure 6:**
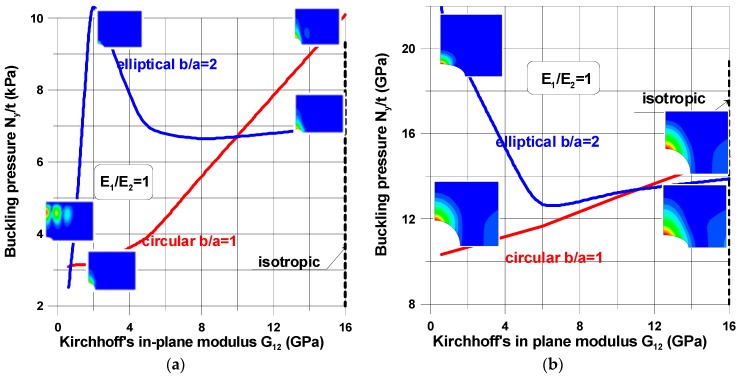
The influence of in-plane shear properties on buckling loads; (**a**) a rectangular plate, *L_x_*/*L_y_* = 1.25, 2*b*/*L_x_* = 0.16; (**b**) a square plate, 2*b*/*L_x_* = 0.4.

**Figure 7:**
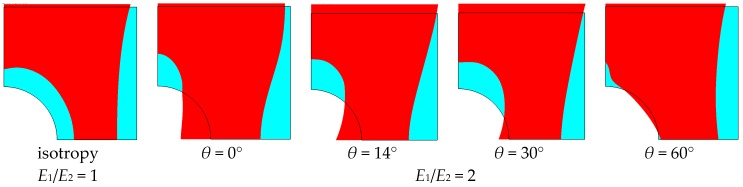
Pre-buckling deformations of the square plate with a circular hole (2*b*/*L_x_* = 0.4). The scaling factor is 5.

**Figure 8:**
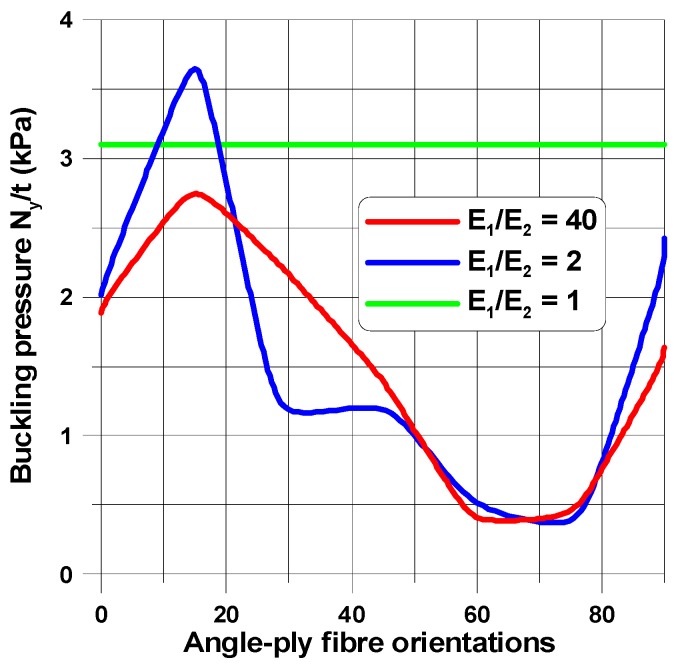
Distributions of buckling loads with fibre orientations (*θ*) and the orthotropic parameter, *β* = *E*_1_/*E*_2_, *G*_12_/*E*_1_ = 0.015*.* The rectangular plate (*L_x_*/*L_y_* = 1.25) for the elliptical horizontal hole, 2*b*/*L_x_* = 0.16.

**Figure 9:**
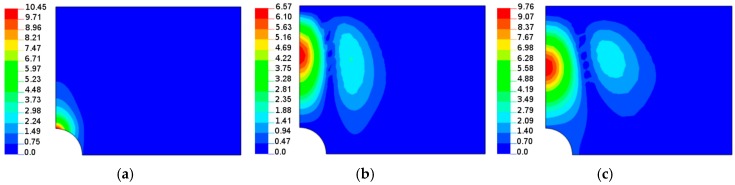
Buckling modes (normal deflections) for the rectangular plate, *L_x_*/*L_y_* = 1.25, with the circular hole 2*b*/*L_x_* = 0.16: (**a**) isotropy *E*_1_/*E*_2_ = 1; (**b**) *E*_1_/*E*_2_ = 2 *θ* = 14°; (**c**) *E*_1_/*E*_2_ = 2 *θ* = 30°.

**Figure 10:**
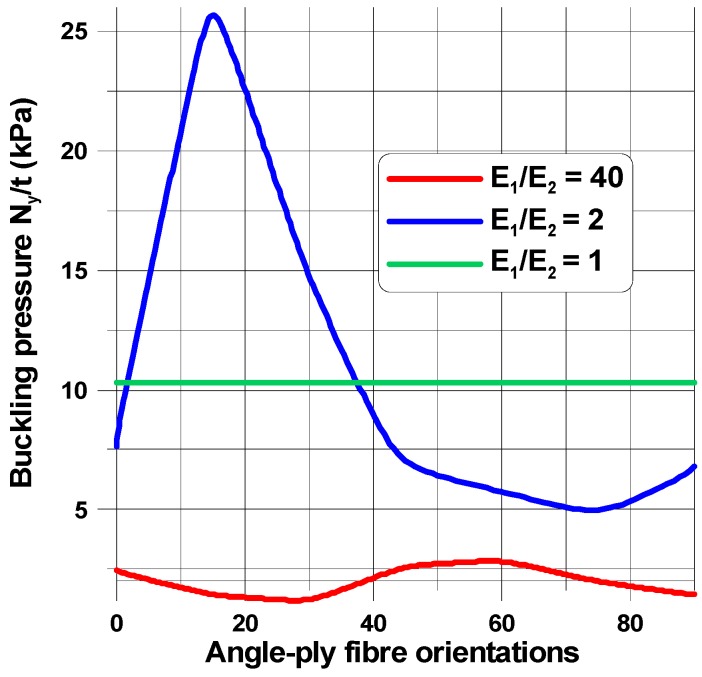
Distributions of buckling loads with fibre orientations (*θ*) and the orthotropic parameter, *β* = *E*_1_/*E*_2_, *G*_12_/*E*_1_ = 0.015 for the square plate with the circular hole 2*b*/*L_x_* = 0.4.

**Figure 11:**
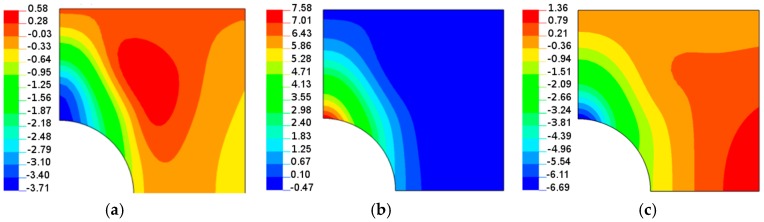
Buckling modes (normal deflections) for the square plate with the circular hole 2*b*/*L_x_* = 0.4: (**a**) isotropy *E*_1_/*E*_2_ = 1; (**b**) *E*_1_/*E*_2_ = 2 *θ* = 14°; (**c**) *E*_1_/*E*_2_ = 2 *θ* = 60°.

**Figure 12:**
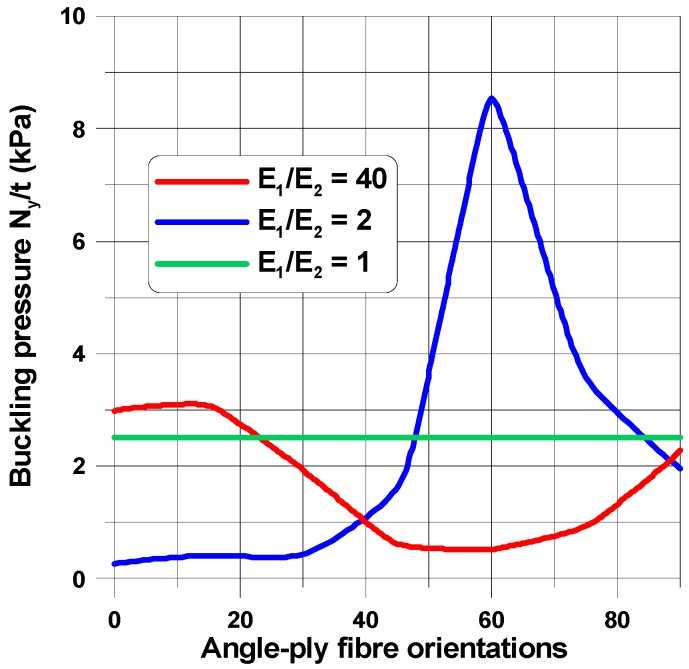
Distributions of buckling loads with fibre orientations (*θ*) and the orthotropic parameter, *β* = *E*_1_/*E*_2_, *G*_12_/*E*_1_ = 0.015 for the rectangular plate (*L_x_*/*L_y_* = 1.25) with the elliptical horizontal hole 2*b*/*L_x_* = 0.16, *b*/*a* = 2.

**Figure 13:**
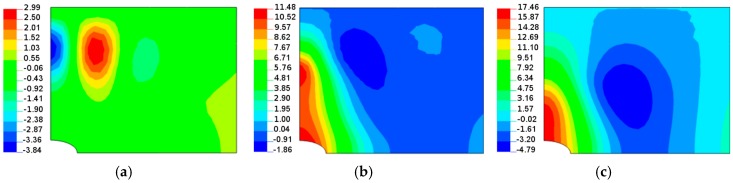
Buckling modes (normal deflections) for a rectangular plate (*L_x_*/*L_y_* = 1.25) with the elliptical horizontal hole, 2*b*/*L_x_* = 0.16, *b*/*a* = 2: (**a**) isotropy *E*_1_/*E*_2_ = 1; (**b**) *E*_1_/*E*_2_ = 2 *θ* = 30°; (**c**) *E*_1_/*E*_2_ = 2 *θ* = 60°.

**Figure 14:**
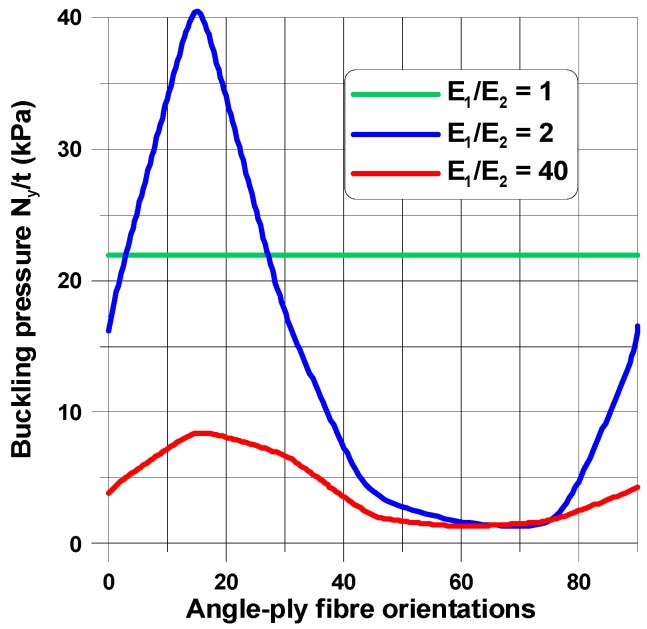
Distributions of buckling loads with fibre orientations (*θ*) and the orthotropic parameter, *β* = *E*_1_/*E*_2_, *G*_12_/*E*_1_ = 0.015 for the square plate with the elliptical horizontal hole 2*b*/*L_x_* = 0.4, *b*/*a* = 2.

**Figure 15:**
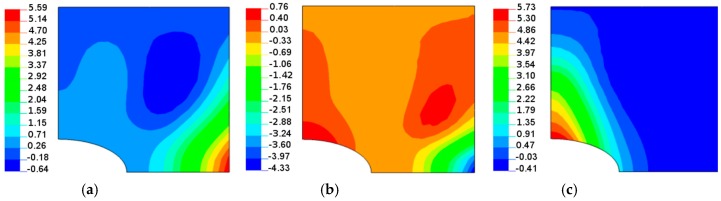
Buckling modes (normal deflections) for the square plate with the elliptical horizontal hole 2*b*/*L_x_* = 0.4, *b*/*a* = 2: (**a**) isotropy *E*_1_/*E*_2_ = 1; (**b**) *E*_1_/*E*_2_ = 2 *θ* = 14°; (**c**) *E*_1_/*E*_2_ = 2 *θ* = 30°.

The buckling load and the shape of buckling mode of the perforated plates are highly influenced by their material properties (Figure 6, Figure 7, Figure 8, Figure 9, Figure 10, Figure 11, Figure 12, Figure 13, Figure 14 and Figure 15). For isotropic plates (the Kirchhoff modulus *G*_12_ = 0.5*E*/(1 + *ν*)), the buckling resistance of plates with elliptical holes (*b*/*a* > 1) is always lower than for plates with circular holes with identical radii (*b*). This effect is different for composite materials where both the in-plane and transverse shear Kirchhoff’s moduli are very low (see Equation (4) and Figure 6). Therefore, to analyse the influence of the value of the orthotropic ratio, *β* = *E*_1_/*E*_2_, on buckling loads, the ratio *G*_12_/*E*_1_ (*G*_12_ = *G*_13_ = *G*_23_) is assumed to be very low and equal to 0.015, e.g., the similar ratio occurs for the graphite epoxy, IM7/8552.

Figure 6 demonstrates the characteristic features of buckling problems and their dependence on both material and geometrical properties. The tensioned panel can buckle in the form of

- a single buckle at the apex of the hole (*x* = 0, *y* = *a*)—the characteristic buckling mode of an infinite tensioned plate;
- two buckles, one localized at the apex and the second around the point *x* = *b*, *y* = 0;
- three buckles located far from a cut-out—it is a characteristic behavior of buckled short tensioned panels [35].

For any values of parameter *β*, the buckling of composite multilayered plates with centrally located cut-outs always affects the major regions of the structure. Next, in this section, in order to show their influence on buckling modes, the angles of fibre orientations (*θ*) are selected in a specific way. In general, we intend to present the possible variations of buckling modes around the maxima of buckling loads.

Figure 1 demonstrates the compressive deformations of plates, but the plots do not show the variation in the pre-buckling displacements with fibre orientations and the orthotropic parameter (see Figure 7). Note that the left edge of the plate is completely free and the bottom one is subjected to symmetry conditions with respect to the x-axis. The maximal displacements, *u_x_* (*u_y_* = 0), occur at the point (0, *L_x_*/2).

When the size of the circular hole is small (comparing to the width and the length of the plate), the compressive stresses are small. These stresses are responsible for the occurrence of buckling (see Figure 8 and Figure 9), but the influence of material properties on buckling loads is not significant since the maximum of the curves plotted in Figure 8 has almost the same value as that of isotropic materials—the difference is about 10–12%. The amplitudes of the buckles are higher than those for laminated structures and take quite a different form (localized at the apex of the hole in the case of isotropic structures). At this point, it is interesting to mention that isotropic and laminated plates show the same wave number in the lateral direction.

As the compression effects increase (the increase of the ratio 2*b*/*L_x_*), the orthotropic effects become much more apparent (Figure 10 and Figure 11). It may be observed by comparison with Figure 10, the initial increase in the pre-buckling deformations (*u_x_*) at the plate corner is associated with a rapid increase in the buckling loads, whereas the further reduction of buckling loads with an increase in the value of *θ* is connected with the decrease in deformations, both at the corner and at the apex of the circular opening, i.e., at *x* = 0, *y* = *a*. For both isotropic and laminated plates, the buckling mode is localized at the apex of the hole (the highest amplitude), and then, the amplitudes of the buckles decrease rapidly in the lateral direction.

Both the buckling loads (Figure 10) and buckling modes vary (Figure 9) with fibre orientation in as similar manner as that observed for the pre-buckling in-plane displacements (Figure 7). It is worth noting that for the lower buckling loads (*θ* > 14°), two waves of buckling appear (Figure 11c) as a result of the plate finite dimensions (compare with the results presented by Kremer, Schurmann [26] for infinite plates).

The change in the shape of the hole from the circular to the elliptical cut-out leads to different distributions of buckling loads with fibre orientations (Figure 8, Figure 10 and Figure 12) since for the horizontal elliptical holes, the compression effects are much higher than for circular holes with identical geometrical ratios: 2*b*/*L_x_* and *L_x_*/*L_y_* (see Tan [33]). Therefore, for elliptical horizontal cut-outs, a reduction of the buckling load values is observed.

Now, the maximal buckling loads occurs at *θ* = 60°. Note that the position of the maximum is shifted from the angle *θ* = 14° (see Figure 8). Note that for the circular cut-out (Figure 8), the local maximum occurs at *θ* = 90° which has a similar value as that for the angle *θ* = 14°. For laminated plates, the maximal amplitudes of the buckling displacements are very high comparing to the circular holes (see Figure 13). They are strictly localized at the top of ellipses and smeared out over the wide area of the hole. In this way, those regions may be the origin of possible first-ply-failure of laminates.

For laminated plates with the horizontal elliptical cut-out (the same *b*/*a* = 2 as previously) with the plate aspect ratio, *L_x_*/*L_y_* = 1, and the plate-width ratio, 2*b*/*L_x_* = 0.4, the distributions of buckling in-plane loads (Figure 14) almost resemble the plots in Figure 8 and Figure 10 (circular holes), and the buckling modes (Figure 15) are quite different than for circular holes. For ellipses at the point (0, *a*), the curvature is higher than that for circular holes, and the number of buckling waves increases to 3. Then, for higher values of the angle (*θ*), it becomes much more localized.

In the comparison with the case of circular holes, due to the increase in the curvature at the apex (0, *a*), an increase in buckling loads is observed. Similar effects were noticed by Kremer and Schurmann [26].

The discussion of the results has been carried out for plates with orthotropic properties. However, the application of composite materials should be estimated by a comparison with the isotropic structures. For all cases considered herein, the applicability of laminated constructions is strictly limited. Useful information can be obtained by the analysis of the variation in buckling loads with different fibre orientations for symmetric angle-ply laminates. The analysis should be always supported by a finite element investigation.

### 4.2. Influence of Plate and Cut-Out Geometry

**Figure 16:**
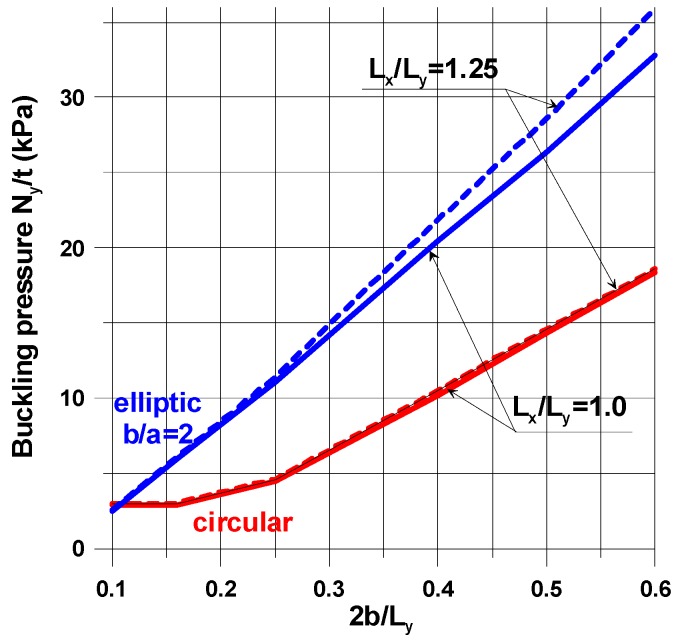
Variation in buckling loads with different geometrical parameters—isotropy *E*_1_/*E*_2_ = 1, *G*_12_/*E*_1_ = 0.015.

For prescribed material properties, the values of buckling loads are also sensitive to the plate aspect ratio, *L_x_*/*L_y_*, and to the plate-width ratio, 2*b*/*L_x_*, that, briefly speaking, characterize the magnitude of the stress concentration effects at the point *x* = *b*, *y* = 0. As the stress concentration effect decreases (2*b*/*L_x_*→0), the values of the buckling loads decrease for both circular and elliptical cut-outs (Figure 16).

## 5. Comparison of Theoretical Predictions and the Finite Element Analysis

A comparison of the analytical and numerical buckling loads of isotropic plates is presented in Table 1. It is assumed that: *E* = 40 (GPa), *ν* = 0.25, *t* = 0.01 (mm), *b* = 20 (mm), and *G*_12_ = 0.5*E*/(1 + *ν*). Note that for horizontal elliptical holes, the radius of curvature increases at *x* = 0 and is equal to *b*^2^/*a*. For square plates with a circular hole, the Kremer approximation [26] seems to be too conservative; however, the results can be derived for infinite plates, i.e., 2*b*/*L_x_* tends towards zero. Shimizu [23] took into consideration the finite width, L_x_, of the plate, and his estimation (Equation (2)) relates better to the FE results, particularly for small holes and rectangular plates. The decrease of the radius of the curvature at the plate apex leads to the reduction of the buckling but the value computed with the use of Relationship (1) are too low. The last row in Table 1 demonstrates the results for an elliptical hole with the same curvature radius as that described previously, but the ratio,2*b*/*L_x_*, is lower. It is not reflected by the analytical predictions.

Strength analyses of stretched composite plates with elliptical cut-outs (see Tan [33], Srivastava [36]) have proven that the correct description of the problem should incorporate three geometrical ratios (*t*/*b*, 2*b*/*L_x_* and *L_x_*/*L_y_*). However, it is impossible to characterize the influence of material properties on the buckling resistance with the use of simple analytical formulas. The above problems have also been discussed by Muc et al. [37,38,39] and Seif and Kabir [34].

## 6. Concluding Remarks

Several key findings and behavioral characteristics were discussed. These findings included the effects of the cut-out size, shape, plate aspect ratio, and orthotropic ratio.

It has been observed that the buckling patterns of stretched plates with holes can be different and cannot be limited to the case of a single buckle around the apex of the hole in the direction of a tensile load. For the lowest buckling load, the existence of two or three buckles is also possible. This evidently demonstrates the origin of the difficulties in the analytical estimations of buckling loads with the use of the Rayleigh quotient or the Bubnov–Galerkin method.

The buckling load is very sensitive to the variations in the material properties, i.e., the *E*_1_/*G*_12_ and *E*_1_/*E*_2_ ratios, due to their significant influences on the pre-buckling deformations and complicated shapes of buckling modes. In the classical analysis of isotropic structures (*G*_12_ = 0.5*E*/(1 + *ν*)), the buckling load of circular holes is always higher than that for elliptical cut-outs. The decrease in the *E*_1_/*G*_12_ ratio (orthotropic composite materials) may lead to an increase in buckling loads for horizontal elliptical holes; they may be higher than those for circular holes (see Figure 16). Changesin the value of the orthotropic ratio, *β* = *E*_1_/*E*_2_, can reduce or increase the value of buckling loads. Thus, it is obvious that the optimal design of material properties is required and recommended.

To solve the above problem, in a general manner, two new design variables were introduced to allow us to analyse the effects of material properties, fibre orientations, and stacking sequences on variation in the buckling loads. The post map method was proposed as a useful tool to capture those effects in a consistent and explicit way. For plates made of plies with identical thicknesses and material properties, it seems that the maximal buckling loads can be reached at the boundaries (the parabola or the polygon) of the feasible region of the defined design variables; the boundaries correspond to angle-ply symmetric fibre orientations.

Since experimental research shows the simultaneous existence of different failure modes (see e.g., Muc et al. [37,38,39]), the detailed first-ply failure (FPF) and delamination (the use of fracture criteria) analysis should be carried out to determine and optimize the dominant failure modes (buckling, FPF or delamination) for perforated laminated plates or shells. Such an analysis should precede the experimental verification of buckling loads for stretched laminated panels.

## Figures and Tables

**Table 1:** Analytical estimations of buckling loads versus finite element computations.

Form of Plate and Shape of Hole	Method of Analysis		
	Kremer [26] Equation (1)	Shimizu [23] Equation (2)	Present FE
Rectangular plate, circular hole, 2*b*/*L_x_* = 0.4, *L_x_*/*L_y_* = 1.25	35.5	10.87	10.19
Square plate, circular hole, *2b*/*L_x_* = 0.4	35.5	10.87	14.94
Square plate, horizontal elliptical hole, 2*b*/*L_x_* = 0.4, *b*/*a* = 2	35.5/4 = 8.88	Not available	13.88
Rectangular plate, elliptical hole, 2*b*/*L_x_* = 0.16, *b*/*a* = 2, *L_x_*/*L_y_* = 1.25	8.88	Not available	35.55

**Figure A1:**
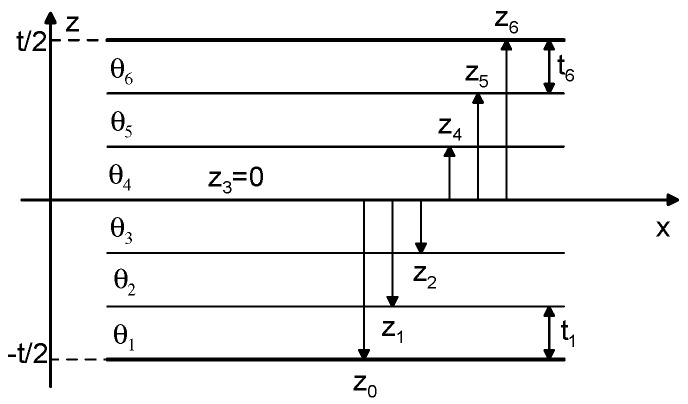
The laminate cross-section (*N* = 6).

## Appendix A

In the local Cartesian system of coordinates the physical relation between the moments *M*_11_, *M*_22_, *M*_66_ and the changes of curvatures *ε*_11_, *ε*_22_, *ε*_12_ (see Equation (3)) can be expressed as follows:

$$\left[\begin{array}{c} M_{11}\\ M_{22}\\ M_{66} \end{array}\right]=\left[\begin{array}{ccc} D_{11} & D_{12} & 0\\ D_{12} & D_{22} & 0\\ 0 & 0 & D_{66} \end{array}\right]\left[\begin{array}{c} \varepsilon_{11}\\ \varepsilon_{22}\\ \varepsilon_{66} \end{array}\right],$$

where

$$\begin{array}{l} D_{11}=\frac{t^{3}}{12}\left(U_{1}-U_{3}+U_{2}x+2U_{3}y\right),\;D_{12}=\frac{t^{3}}{12}\left(U_{4}+U_{3}-2U_{3}y\right),\\ D_{22}=\frac{t^{3}}{12}\left(U_{1}-U_{3}-U_{2}x+2U_{3}y\right),\;D_{66}=\frac{t^{3}}{12}\left(U_{5}+U_{3}-2U_{3}y\right), \end{array}$$

$$U_{1}=\frac{1}{8}(3Q_{11}+3Q_{22}+2Q_{12}+4Q_{66}),\;U_{2}=\frac{1}{2}(Q_{11}-Q_{22}),\;U_{3}=\frac{1}{8}(Q_{11}+Q_{22}-2Q_{12}-4Q_{66}),$$

$$U_{4}=\frac{1}{8}(Q_{11}+Q_{22}+6Q_{12}-4Q_{66}),U_{5}=\frac{1}{2}(U_{1}-U_{4}),$$

$$Q_{11}=\frac{E_{1}}{1-\nu_{12}\nu_{21}},Q_{12}=\frac{\nu_{12}E_{2}}{1-\nu_{12}\nu_{21}},Q_{22}=\frac{E_{2}}{1-\nu_{12}\nu_{21}},Q_{66}=G_{12},$$

The variables *x* and *y* denote the design variables and are defined in the following way:

$$x=\frac{4}{t^{3}}\sum_{l=1}^{N}\left(z_{{}_{l}}^{3}-z_{{}_{l-1}}^{3}\right)\cos(2\theta^{\left(l\right)}),\;y=\frac{4}{t^{3}}\sum_{l=1}^{N}\left(z_{{}_{l}}^{3}-z_{{}_{l-1}}^{3}\right)\cos^{2}(2\theta^{\left(l\right)}),\;$$

*z_l_* and *z_l−_*_1_ are the location coordinates of the top and the bottom surfaces of the lamina, *l* (see Figure A1).

The above definitions are valid both for continuous and discrete fibre orientations. However, for discrete fibre orientations, it is much more convenient to use specific coding and decoding procedures, as described by Muc [40].
